# Human Immunodeficiency Virus (HIV)-1 Integration Sites in Viral Latency

**DOI:** 10.1007/s11904-014-0241-9

**Published:** 2015-01-09

**Authors:** Simin D. Rezaei, Paul U. Cameron

**Affiliations:** 1 Faculty of Medicine, Dentistry and Health Sciences, Department of Microbiology and Immunology, Doherty Institute for Infection and Immunity, The University of Melbourne, 4th Floor, 786-798 Elizabeth St, Melbourne, 3010 Australia; 2 Infectious Diseases Unit, Department of Infectious Diseases, Alfred Hospital, 85 Commercial Rd, Melbourne, Victoria 3004 Australia

**Keywords:** HIV, Integration sites, Latency, Jurkat cell line, CD4 T cells, Transcriptional units, Transcriptional interference, Cancer-associated genes, T cell clonal expansion, T cell subsets

## Abstract

The persistence of human immunodeficiency virus type 1 (HIV-1) in latent reservoirs is a major barrier to HIV cure. Reservoir establishment depends on low viral expression that may be related to provirus integration sites (IS). In vitro, in cell lines and primary T cells, latency is associated with specific IS through reduced viral expression mediated by transcriptional interference by host cellular promoters, reverse orientation, and the presence of specific epigenetic modifiers. In primary T cell models of latency, specific IS are associated with intracellular viral antigen expression that is not directly related to cell activation. In contrast, in patient CD4+ T cells, there is enrichment for IS in genes controlling cell cycle and survival and in some clonally expanded T cell subpopulations. Multiple insertion sites within some specific genes may suggest that integrated HIV can increase the host’s T cell survival.

## Introduction

Despite combination antiretroviral therapy (cART) that can effectively control viral replication and normalize immune function, human immunodeficiency virus (HIV) remains a global health issue. Stopping therapy is almost invariably associated with recurrence of HIV viremia because of HIV persistence in latent reservoirs that remain one of the major barriers to HIV cure [1].

The latent viral reservoir exists as an immunologically undetected pool of infection in patients on cART [2–4]. The latent reservoir is established early during infection [5, 6] and latent proviruses can be found, albeit at different frequency, in all CD4+ T cell subsets including naive (T_NA_), stem cell memory (T_SCM_), central memory (T_CM_), effector memory (T_EM_), and terminally differentiated (T_TD_) T cells [7–10] as well as in monocyte and macrophages [11–13]. The ability of HIV-1 to generate reservoirs in different cell subsets leads to the formation of a heterogeneous population of infected CD4+ T cells with different life span, different frequency of viral latency, and potentially different requirement for cellular and viral activation [10, 14]. It is suggested that the heterogeneity of the reservoir may play a role in stability and persistence of latency [14–16].

Although latent HIV is defined as replication-competent but transcriptionally silent infection, only some of the full-length intact proviruses can be induced following cellular stimulation [17, 18, 19•], and there is a pool of integrated provirus that is apparently noninducible. Activation of these pools of latent HIV-1 by latency reversing agents (LRA) that induce viral expression is one of the cornerstones of strategies to eliminate latency by “kick and kill.”

Study of the cellular and molecular establishment of the latent reservoir has used in vitro models of latency in cell lines and primary CD4+ T cells and analysis of residual HIV in resting CD4+ T cells from HIV-infected patients on cART. It is well established that HIV-1 integration sites (IS) are not randomly distributed in the genome and differ in preferred genomic sites from other retroviruses [20]. The regulation of integration site selection may be critical for understanding HIV latency and the potency of LRA in eliminating HIV, particularly for those that effect epigenetic modification. In this review, we focus on understanding the effect of HIV-1 integration in the in vitro models of latency and the recent data identifying some IS that may directly determine the persistence of latency either by favoring infected cell survival or by maintaining low viral expression.

### In Vitro HIV-1 Integration Occurs Predominantly Within Transcriptional Units Outside Promoter Regions

Initial studies of genomic distribution of HIV-1 provirus in T cell lines found IS in gene-rich regions [21–23] (Table 1). Integration of provirus at sites of active transcription promotes viral expression, while integration in the site with low level of transcription can delay proviral transcription and promote latency [23]. One of the major elements regulating transcription is host cell chromatin. The fundamental structure of chromatin is the nucleosome, which contains repeating histone-containing units including H2A, H2B, H3, and H4 [24]. Further analysis showed that the site of HIV-1 integration can remodel nucleosome via changes in histone acetylation at the site of integration or via recruitment of specific chromatin remodeling like histone deacetylase inhibitors (HDACi) increasing transcription activity [24]. These suggested a correlation between proviral IS and epigenetic modifications of chromatin environment at the site of integration. This notion was further supported by studies on molecular mechanism of IS in host genome [25, 26]. It was shown that depletion of chromatin reassembly factors (CRFs) like Spt6, Chd1, FACT as well as the histone chaperons ASF1a and HIRA at the site of integration resulted in chromatin relaxation promoting proviral expression [27]. This highlighted that the presence of these CRFs influences the accessibility of the transcription factors to HIV promoter, blocking the elongation process leading to formation of latency through transcriptional interferences [26, 27].

**Table 1:** Studies of HIV-1 integration sites (IS) in vitro and in vivo

Type of study	Cell type	No. of IS	Location	Main observations	Reference
In vitro infection of T cell lines					
T cell line	Jurkat	7171	GDR	IS within gene dense regions	[44]
T cell line	J-Lat A1	130	NP	Inducible provirus in gene deserts	[45]
T cell line	Jurkat	40,569	TAHM	IS within genes active in mitosis, life cycle, and RNA metabolism	[25]
T cell line	Jurkat	971	α-Repeat, GD	IS in low expression genes	[76]
T cell line	Hu primary cells and cell lines	10	TSS	Latency in IS in reverse orientation compared to host gene	[77]
Cell line	Hu primary cells and cell lines	3127 MLV, HIV, ASLV	TU	75 % of integration into tissue-specific active genes	[20]
T cell line	Jurkat	8	Heterochromatin	IS within genes with low level of transcription	[23]
Cell line	HeLa and H9	903 MLV 379 HIV	TU	TSS favored by MLV not by HIV	[30]
T cell line	SupT1	59	TU	IS within genes with high gene density and GC-rich regions	[22]
T cell line	SupT1	524	GDR	IS within introns and alpha satellite	[21]
Cell lines	Jurkat, HeLa, 293T, SW13, CEM, and A301	34	NR	Specific IS determine the expression of latent proviruses	[24]
T cell line	SupT1	61	TU	IS not near centromere	[46]
In vitro infection of primary T cells					
Primary cells	Activated and rCD4 T cells infected by spinoculation	6184 inducible 6252 express	Away from CpG, α-repeat heterochromatin	Latency in low expression genes. EGFP HIV similar in resting or activated. No consistent IS across models	[66••]
Primary cells	Activated and rCD4 T Cells	NR	α-Repeat, GC region	Integration site selection promotes viral expression	[64•]
Primary cells	Bcl2_Transduced CD4 T Cells	224 Ac 216 Ch 493 lat	TU and HAG	Integration sites in same orientation. Transcriptional interference lead to latency	[19•]
Primary cells	Activated and rCD4 T cells	451	TSS and GC regions	IS were selective	[53]
Primary cells	Activated and rCD4 T cells	2661	TU and AG	IS were similar in activated vs resting CD4 T cells	[65]
Primary cells	PBMC and monocytes	754	TU	IS were different in macrophages compared to dividing PBMC	[54]
Integration sites in vivo and patient-derived T cells					
Patients on cART	PBMC, CD4 T cells	1632	Growth and cell regulation	Clonal expansion of HIV-infected cells, multiple IS in BACH2 and MKL2	[68••]
Patients on cART	Unsorted PBMC	534	Proliferation and survival	Multiple insertion in 12 cancer-related genes including BACH2	[69••]
Patients on cART	PBMC, rCD4 T cells	594	TU	Clonally expanded cells, multiple IS in SMC5 and BACH2 genes	[71•]
Patients on cART	rCD4 T cells	62	TU	92.9 % of noninduced provirus found in active transcribed units	[67••]
Patients on cART	rCD4 T cells	457	TU	Selective integration in BACH2	[72]
Untreated patients	PBMC, tissues	43	GDR	Viral integration in coding genes	[78]
In vivo	CD34+ from rhesus macaque	432 MLV 328 SIV	TU, SIV, TSS, MLV	Site around TSS favored by MLV, but IS within the TU in SIV	[29]
Patients on cART	rCD4 T cells	74	TU	IS in actively transcribed genes	[52]

*GDR* gene dense region, *NP* nuclear periphery, *TAHM* transcriptional associated histone modification, *GD* gene dessert, *TSS* transcription start site, *TU* transcriptional unit, *NR* not reported, *rCD4* resting CD4 cells, *HAG* highly active genes, *AG* active gene, *PBMC* peripheral blood mononuclear cells, *MLV* murine leukemia virus, *ASLV* avian sarcoma-leukosis virus, *Ac* acute, *Ch* chronic, *lat* latent, *pat* patient

### HIV-Specific Determinants of Integration Sites

Comparative studies of IS in retroviral infection with murine leukemia virus (MLV), HIV, and avian sarcoma-leukosis virus (ASLV) showed that the IS selection differs among retroviruses [28, 29]. HIV-1 predominately targets transcriptional unit (TU) of active genes, while MLV and ASLV favor transcription start sites (TSS), particularly regions in promoters and upstream of start codons that are uncommon IS for HIV [20, 30]. The differences between IS selections among retroviruses (ASLV, MLV, and HIV) have been attributed to the interaction between viral-associated integrase enzyme and host cellular factor LEDGF/p75 [31]. The cellular protein lens epithelium-derived growth factor (LEDGF) and coactivator protein p75 have shown to form a stable tetramer structure with HIV-1 viral integrase enzyme [31]. This structure forms at regions rich in 5′ GT (A/T) AC 3′ [32], enhancing strand transfer activity of viral integrase. In addition, depletion of LEDGF/p75 led to loss of the preferential integration of HIV-1 into TU of the host genome [33] and replacing LEDGF/p75 chromatin interaction domain by fusion proteins resulted in redirecting the HIV-1 IS in G/C-rich regions [32, 34, 35]. The role of host cellular factor in viral integration was further supported by substitution of LEDGF/p75 with hepatoma-derived growth factor-related protein 2 (HRP2) [35, 36••], where the conserved pro-trp-trp-pro (PWWP) domain common in both proteins could bind to modified histone leading the proviral integration into active TU [37•]. In contrast, in MLV, the association between integrase enzyme and LEDGF/p75 was weaker, which might explain the different preferences of integration site selection in these two viruses [28]. Subsequently, the interaction of viral integrase enzyme with several other cellular proteins including barrier-to-autointegration factor (BAF) [38, 39] and high-mobility-group family 1 (HMG 1 Y) [40, 41] was also described. These two cellular proteins are small DNA binding proteins, which are able to modify DNA structure at the site of integration and increase the probability of integration. Collectively, these data suggested interaction between host cellular proteins and HIV-1 integrase enzyme resulting in chromatin remodeling at the site of integration and selectively favoring proviral integration into actively transcribed genes [42, 43]. Although preferential integration of HIV-1 provirus in transcriptionally active regions was supported in subsequent studies [20, 25, 44], the transcriptionally silent, but replication-competent proviruses were also reported in regions with low level of transcription including gene dessert [45] and alphoid regions in heterochromatin [46].

### HIV Integration Site Can Determine Expression In Vitro

In the Jurkat, T cell line, there is a relationship between site of integration with viral expression and viral latency. The integration of provirus in actively transcribed gene resulted in an efficient transcription of viral proteins, while integration in sites with low transcriptional activity would result in delay in viral expression or latency [24]. The critical factors appear to be the position and the orientation of the provirus in relationship to actively transcribed genes as well as the methylation of CpG [19•, 27, 42].

#### Orientation of Provirus in IS

The effect of orientation of the provirus in promoting latency was supported by subsequent studies, where HIV-1 provirus integrated into active genes, orientation relative to the host genes could increase the HIV-1 transcription by >10 fold, while integration at opposite orientation reduced the HIV-1 gene expression by 4-folds [47]. These observations highlighted that low level of viral expression is not because of lack of transcriptional activity at the site of proviral integration and it might be due to the presence of factors blocking the transcription at the site of integration by transcriptional interferences (TI) [19•, 47–49].

#### Transcriptional Interference

TI occurs when transcription from the host promoter prevents the transcription of the provirus at the 5′ long terminal repeat (LTR) region downstream of the transcribed gene [50]. The effect of TI has been shown previously in ALV virus, when active transcription from the 5′ LTR was able to block transcription from the 3′ LTR region [51]. Therefore, in TI, there is an ongoing transcription activity from the upstream promoter [19•, 22, 52–54]. TI can inhibit the expression of viral proviruses and promote latency through promoter competition caused by neighboring genes. It has shown that when HIV-1 provirus integrated in a face to face position with the host gene promoter (i.e., in a convergent position), the assembly of the initiation complex necessary for transcription is blocked by promoter occlusion resulting in silencing of the HIV-1 promoter [50]. It is clear that the virus integrated into the gene in the reverse orientation is transcribed at a low level via transcriptional interferences [19•, 27]. Therefore, it is likely that the relationship of the IS and the transcriptional activity of the host gene can shift the balance from proviral expression toward latency.

### HIV Integration Site Analysis in Primary T Cells

HIV-1 latency can be generated through postactivation and preactivation pathways. Postactivation latency refers to direct infection of activated CD4 T cell population, where viral production results in depletion of infected cells, but some revert to a resting stage containing HIV-1-integrated provirus [55]. The integrated proviruses generated through this path are commonly replication competent; however, due to some epigenetic modifications at the site of integration, they become transcriptionally silent [56]. Infection of resting cells may result in preintegration or postintegration latency. Preintegration latency occurs after direct infection of resting CD4 T cells and incomplete reverse transcription or a block at steps prior to integration [57]. This transient infection may be rescued by TcR ligation or mitogen activation resulting in the viral antigen expression and virus production [58, 59]. If viral expression is blocked after integration, a postintegration form of latency, we have called preactivation latency, can be generated. This occurs in vitro by direct infection of chemokine-treated resting CD4 T cells [60, 61], by spinoculation or by co-culture with dendritic cells [62] or endothelial cells [63].

#### Integration in Resting and Activated T Cells

Several studies have examined integration in latently infected cells by direct infection of resting and activated CD4+ T cells in vitro [53, 64•, 65, 66••]. The main differences of infected resting cells compared to productively infected activated CD4+ T cells were an increase in defective sequences in the LTR, and an increase in 2LTR circles (a by-product of virus that fails to integrate) was observed in resting cells [53]. HIV IS have been examined following spinoculation of resting CD4+ T cells infected with green enhanced fluorescent protein (EGFP) expressing HIV. In cells expressing low levels of EGFP but not producing free virus, the IS differed from latently infected cells that had no EGFP expression [64•]. In the most extensive study of integration sites in silent and productive sites in primary T cells in five different models of latency [66••], the epigenetic modifiers at the site of HIV integration even when in an actively transcribed gene had only a modest effect in determining the level of expression, and no consistent features could be demonstrated across the different models.

### HIV Integration Site Analysis in Patient-Derived Cells

One of the main difficulties with the analysis of HIV IS in patient-derived samples is the problem to identify replication-competent proviruses in latently infected cells. Ho et al. have shown that only a small proportion (<1 %) of the integrated HIV are induced following cellular reactivation [67••]. However, their analysis showed the presence of a large unmeasured hidden population of potentially replication-competent noninducible proviruses (11.7 %) in the latent reservoir [67••], of which 92.9 % were found in the transcription-active unit. These proviruses had an intact genome, with unmethylated promoter, and the possible potential to express infectious virus following cellular activation. How replication competent, but transcriptionally silent provirus can persist in patients on therapy is still unclear. Although hemostatic proliferation of latently infected cells has been proposed as a crucial factor for persistence of the viral reservoir [16], two recent studies have highlighted the role of IS in well-suppressed patients.

### Enrichment of IS in Genes Regulating Cell Growth and Specific Cancer-Associated Genes

In the study by Maldarelli et al., a total of 2410 IS isolated from five patients were analyzed [68••], of which 43 % (1022) of IS were associated with more than one host gene. This showed that a large population of infected cells was generated from expansion of a single clone. In one out of five patients tested in this study, 20 % of integration events (i.e., 62 out of 317) were found in the same site in HORMAD2 gene (64 integrants (INT)). This analysis showed that almost 58 % of IS in patients were derived from a single HIV-1-infected cell. Subsequent analysis showed that approximately 70 % (21 of 29) of genes targeted more than once were known to be directly involved in cell growth (STATB5 (18 INT)) or mitosis (MAP4 (7 INT)) (Table 2). These data suggested clonal expansion of HIV-1-infected cells as a potential mechanism for persistence of HIV-1 reservoir in well-suppressed patients on cART.

**Table 2:** List of host genes selectively targeted by HIV-1 in vivo*.* Genes are indicated by gene symbol (numbers of integrants)

Ikeda_2007^a^	Maldarelli_2014^b^	Wagner_2014^c^	Imamichi_2014^d^
*BACH2 (44)*	*BACH2 (25)*	*BACH2 (16)*	*BACH2 (4)*
**MKL2 (4)**	**MKL2 (122)**	**MKL2 (4)**	***SMC5 (17)***
*STAT5B (16)*	*STAT5B (18)*	*SATA5B (4)*	
SMG1 (9)	CYTH1 (7)	**XAF1 (13)**	
RARRES3 (3)	RPTOR (7)	**ARHGP35 (7)**	
ZGPAT (2)	TAOK1 (8)	*CREBBP (3)*	
EIF4G (3)	TNRC6B (7)	**MOB1A (2)**	
SP192 (2)	PRKCB (7)	**MGA (3)**	
hmRNA (8)	MKL1 (9)	**GPC3 (2)**	
SPTANI (2)	***MAP4 (7)***	*CBL (2)*	
SPATS2 (4)	PAK2 (10)	PHF (9)	
TOP1 (2)	NSD1 (7)	APOBEC3C (15)	
DUSP16 (2)	KIAA0319L (12)	*MDC1 (57)*	
SOX5 (4)	NFATC3 (7)	HSF5 (10)	
NRF1 (2)	***HORMAD2 (64)***	RALGPS2 (7)	
	BTBD9 (6)	OXCT1 (5)	
	UBE2H (8)	SMG6 (14)	
		TBCD (14)	

Genes associated with cell growth and cell proliferation (italics), genes associated with mitosis (bold italics), genes associated with cancer (bold)

^a^Longitudinal analysis of IS in seven patients on therapy (>2 years), detected in each gene

^b^Longitudinal analysis of IS in five well-suppressed patients on cART (>10 years); only genes with >5 integrants are included in the table

^c^Longitudinal analysis of IS in three well-suppressed patients on cART (>10 years)

^d^Longitudinal analysis of IS in one patient on therapy (≥15 years)

In a separate study by Wagner et al., a total of 534 integrated proviruses isolated from three patients were analyzed and the proviral IS was found with the same location in multiple cells within each patient. Identical IS were derived from ≥2 individual cells, thus highlighting the proliferation of infected cells in these patients [69••]. Subsequent analysis on the proviral site of integration in this population revealed preference of integration in cancer-associated genes. Among 1332 unique genes examined for overrepresentation of IS, 12.5 % (36/288) were cancer-associated genes. In a comparative analysis between IS derived from patient samples with those from acutely infected Jurkat T cell line, a similar pattern of integration was observed in cancer-related genes (12.70 vs 11.4 %, respectively). However, IS detected in proliferating cells derived from patients were enriched in cancer-associated genes compared to IS detected in Jurkat cells (i.e., 15.97 vs 11.14 %, respectively). The genes like CREBBP, STAT5B, BACH2, C2CD3, and MKL2 (Table 2) were found in two or three participants tested in this study, suggesting preferential integration of HIV-1 provirus in these regions. These observations raised the possibility of the effect of integration site on altering the function of these genes favoring long-term survival.

### Viral Gene Expression and Latent Infection In Vivo

In the early stages of HIV-1 infection, there is a high turnover of both virus and infected cells [70] leading to activation of adaptive immune system and subsequent clearance of the infected cells [4]. Differential clearance of virally infected cells will lead to accumulation of cells that do not express viral proteins and evade immunological recognition or have no cytopathic loss. Both pathways of loss will depend on defective provirus or HIV latency. The in vitro models of latency have characterized factors affecting proviral expression including orientation of the provirus [19•] with neighboring host genes as well as TI [47] and epigenetic modifiers. In the study by Maldarelli et al., multiple proviruses were found in MKL2 genes in a patient on suppressive antiviral therapy. The proviruses shared the same transcriptional orientation and located upstream of the start codon of the MKL2 genes. There were also 15 independent IS found in the BACH2 gene. The integrants shared the same orientation with the BACH2 gene and located in introns 4 and 5 of the gene [68••]. A comparative analysis of IS detected in the patient samples with acutely infected HeLa cells and human CD34+-infected cells (in vitro) showed higher frequencies of IS in both MKL2 (7 vs 0.03 % in HeLa and CD34+ cells) and BACH2 (i.e., 1.5 vs 0.002 % in HeLa and 0.01 % in CD34+ cells) in patient samples. The IS were found to be more widely spread and had multiple orientation patterns in acutely infected primary cells and cell line compared to patient samples [68••]. In the study by Wagner et al., multiple HIV-1 proviral DNA were found in the BACH2 gene in two out of three participants tested in the study [69••]. Comparative analysis with previously reported reoccurrence of HIV-1 IS in BACH2 genes in vivo [67••, 71•, 72] showed that all integration events have occurred in intron 5 of the gene, upstream of the start codon and in the same orientation with the BACH2 gene. The integration patterns were distinct from the patterns reported in clonally infected cell line (Jurkat) where they found only one integrant in intron 5 in BACH2. Although the presence of HIV-1 proviruses in the same orientation with host genes might result in transcriptional interferences from the host promoter promoting latency [19•, 50, 71•], however, the questions remain whether these proviruses are truly examples of independent insertional events and if they are replication competent or not.

In addition, a longitudinal study of HIV-1 proviral DNA in an infected individual on therapy revealed the presence of cell-associated HIV-1 RNA transcripts from a replication-incompetent provirus [71•]. The sample was collected over a period of 17 years, and the provirus carried a premature stop codon at position 42 (W42Stop) in HIV protease gene. Integration site analysis showed that the provirus was integrated within the intragenic region, in opposite orientation to the SMC5 gene. The same mutation was observed in an additional four patients. The expression of full viral envelope proteins was detected from all defective proviral RNA, while there was partial, low, or no expression of Gag-Pol proteins compared to wild-type provirus. It is unclear whether the expression of viral proteins from a defective provirus is a result of readthrough transcripts [19•, 47] or epigenetic modifications associated with the site of integration [49]. However, detection of low level of viral proteins from latently infected cells in patients on long-term therapy may contribute to ongoing immune activation and production of cytokines and chemokine and promote homeostatic proliferation of infected cells and maintenance of reservoir [16].

In order to understand the mechanisms for maintaining the latent reservoir, further work is needed to determine whether the latent proviruses in well-suppressed HIV-infected patients are defective proviruses that have selectively accumulated by survival of a particular clone or represent the large pool of unactivatable virus that represents IS within genomic sites that promote temporary latent state.

### Integration Sites in Specific Subsets of CD4 T Cells

Several studies have shown that clonal expansion of infected T cells in patients on long-term therapy is selective and driven by homeostatic proliferation. In the study of 31 aviremic patients, >50 % of cells harboring replication-competent proviruses were detected in memory T cell subsets [16]. Proviruses were mainly found in T_CM_ and transitional memory T cells (T_TM_) but not in T_EM_. Both T cell populations (i.e., T_CM_ and T_TM_) have been shown to have low level of proliferation due to immune activation caused by long-term therapy. These cells also have a long half-life which would help maintain viral reservoir in these subsets [16].

In addition, replication-competent viruses have been detected in CD4 T memory stem cell-like properties (T_SCM_) cells isolated from three patients on continuous treatment with HAART [8]. Much higher levels of HIV-1 DNA occur in CD4 T_SCM_ cells compared to CD4 T_CM_, CD4 T_EM_, and CD4 T_TD_ isolated from patients. The contribution of CD4T_SCM_ to the total size of the reservoir was most pronounced in patients with a small reservoir size. This data suggested HIV-1 in infected CD4 T_SCM_ can persist as a stable viral reservoir. Considering CD4 T_SCM_ subsets are permissive to the viral infection, have a low rate of apoptosis, and can survive for a long period [73], it raises the question of whether there is also selective targeting of this population or differences in IS.

In other T cell populations, long-term persistence may be associated with defective provirus. Such expansion of defective DNA in CD4 T_EM_ subsets was found in a well-suppressed infected individual [71•]. Longitudinal analysis of the integration sites found the same site in 17 independent events, and identical viral sequences carrying W42Stop mutation suggested expansion of this particular subset over 15 years. Effector memory T cells are terminally differentiated T cells with the estimation half-life of 3 to 6 days [74, 75]. Thus, survival of the CD4 T_EM_ in an HIV-1-infected individual over 15 years strongly suggested that the persistence of proviruses in this subset is selected by the lack of viral antigen expression. It will be important now to expand studies of IS in T cell subpopulations isolated from patients on long-term therapy to determine how viral expression may be controlled by particular epigenetic markers and gene-specific control mechanisms in the specific subpopulations that contribute most to the size of the latent reservoir.

## Conclusion

Although correlation of IS with expression in T cell lines has provided some evidence of specific characteristics of the genomic regions that downregulate expression and favor latency and has provided tools such as latently infected cell lines such as J-LAT [59] that can be used in screening for LRA, a clear demonstration of similar associations in primary cells has been more difficult. A correlation between viral antigen expression and specific genomic markers has been shown within different in vitro models of HIV latency, but the correlations have not translated to the general features of IS that extend across multiple models of latency in primary CD4+ T cells. Further, correlation of epigenetic markers with antigen expression across different states of cellular activation limits the usefulness of IS analysis as a predictor for efficacy or selection of LRA in reversing latency. The expansion of specific integration sites in patient CD4+ T cells has provided novel insights into the reservoir of latent HIV in vivo. This expansion is the result of two mechanisms. First the clonal expansion of cells infected with virus identical at both insertion sites and by viral sequence. This follows the observations made with viral sequence analysis alone and raises the question if this IS resides in a site favoring latency or if the virus is noninducible or defective. The second possibility raised by the recent findings of a clear enrichment for near identical or multiple IS in specific sites of a gene is that integration in specific genes provides a survival signal for cells carrying an HIV provirus in specific genes in those cells. That the enriched genes are associated with cell survival and cell cycle raises the intriguing possibility that HIV IS may alter the function of those genes and provide cell survival signals. It will be important now to determine if this is a direct genomic effect or if expression of specific HIV gene products is involved. Are readthrough HIV transcripts involved in controlling gene expression or is this an “oncogene” effect?

## References

[1] Siliciano, R.F. and W.C. Greene, *HIV latency.* Cold Spring Harbor Perspectives in Medicine, 2011. 1(1). 10.1101/cshperspect.a007096 PMC3234450 22229121

[2] Finzi D (1997). Identification of a reservoir for HIV-1 in patients on highly active antiretroviral therapy. Science.

[3] Chun TW (1997). Presence of an inducible HIV-1 latent reservoir during highly active antiretroviral therapy. Proc Natl Acad Sci U S A.

[4] Ho DD (1995). Rapid turnover of plasma virions and CD4 lymphocytes in HIV-1 infection. Nature.

[5] Chun TW (1995). In vivo fate of HIV-1-infected T cells: quantitative analysis of the transition to stable latency. Nat Med.

[6] Stellbrink HJ (1997). Asymptomatic HIV infection is characterized by rapid turnover of HIV RNA in plasma and lymph nodes but not of latently infected lymph-node CD4+ T cells. Aids.

[7] Chun TW (2000). Relationship between pre-existing viral reservoirs and the re-emergence of plasma viremia after discontinuation of highly active anti-retroviral therapy. Nat Med.

[8.•] Buzon MJ (2014). HIV-1 persistence in CD4+ T cells with stem cell-like properties. Nat Med.

[9] Blankson JN, Persaud D, Siliciano RF (2002). The challenge of viral reservoirs in HIV-1 infection. Annu Rev Med.

[10] Lugli E (2013). Superior T memory stem cell persistence supports long-lived T cell memory. J Clin Invest.

[11] Koenig S (1986). Detection of AIDS virus in macrophages in brain tissue from AIDS patients with encephalopathy. Science.

[12] Koppensteiner H, Brack-Werner R, Schindler M (2012). Macrophages and their relevance in human immunodeficiency virus type I infection. Retrovirology.

[13] Kumar A, Abbas W, Herbein G (2014). HIV-1 latency in monocytes/macrophages. Viruses.

[14] Chomont N (2011). Maintenance of CD4+ T-cell memory and HIV persistence: keeping memory, keeping HIV. Curr Opin HIV AIDS.

[15] Bosque A (2011). Homeostatic proliferation fails to efficiently reactivate HIV-1 latently infected central memory CD4+ T cells. PLoS Pathog.

[16] Chomont N (2009). HIV reservoir size and persistence are driven by T cell survival and homeostatic proliferation. Nat Med.

[17] Chun TW (2003). Gene expression and viral production in latently infected, resting CD4+ T cells in viremic versus aviremic HIV-infected individuals. Proc Natl Acad Sci U S A.

[18] Hermankova M (2003). Analysis of human immunodeficiency virus type 1 gene expression in latently infected resting CD4+ T lymphocytes in vivo. J Virol.

[19.•] Shan L (2011). Influence of host gene transcription level and orientation on HIV-1 latency in a primary-cell model. J Virol.

[20] Mitchell RS (2004). Retroviral DNA integration: ASLV, HIV, and MLV show distinct target site preferences. PLoS Biol.

[21] Schroder AR (2002). HIV-1 integration in the human genome favors active genes and local hotspots. Cell.

[22] Elleder D (2002). Preferential integration of human immunodeficiency virus type 1 into genes, cytogenetic R bands and GC-rich DNA regions: insight from the human genome sequence. FEBS Lett.

[23] Jordan A, Bisgrove D, Verdin E (2003). HIV reproducibly establishes a latent infection after acute infection of T cells in vitro. Embo j.

[24] Jordan A, Defechereux P, Verdin E (2001). The site of HIV-1 integration in the human genome determines basal transcriptional activity and response to Tat transactivation. Embo J.

[25] Wang GP (2007). HIV integration site selection: analysis by massively parallel pyrosequencing reveals association with epigenetic modifications. Genome Res.

[26] Pearson R (2008). Epigenetic silencing of human immunodeficiency virus (HIV) transcription by formation of restrictive chromatin structures at the viral long terminal repeat drives the progressive entry of HIV into latency. J Virol.

[27] Gallastegui E (2011). Chromatin reassembly factors are involved in transcriptional interference promoting HIV latency. J Virol.

[28] Derse D (2007). Human T-cell leukemia virus type 1 integration target sites in the human genome: comparison with those of other retroviruses. J Virol.

[29] Hematti P (2004). Distinct genomic integration of MLV and SIV vectors in primate hematopoietic stem and progenitor cells. PLoS Biol.

[30] Wu X (2003). Transcription start regions in the human genome are favored targets for MLV integration. Science.

[31] Cherepanov P (2003). HIV-1 integrase forms stable tetramers and associates with LEDGF/p75 protein in human cells. J Biol Chem.

[32] Ciuffi A, Bushman FD (2006). Retroviral DNA integration: HIV and the role of LEDGF/p75. Trends Genet.

[33] Llano M (2004). LEDGF/p75 determines cellular trafficking of diverse lentiviral but not murine oncoretroviral integrase proteins and is a component of functional lentiviral preintegration complexes. J Virol.

[34] Ferris AL (2010). Lens epithelium-derived growth factor fusion proteins redirect HIV-1 DNA integration. Proc Natl Acad Sci U S A.

[35] Gijsbers R (2010). LEDGF hybrids efficiently retarget lentiviral integration into heterochromatin. Mol Ther.

[36.••] Schrijvers R (2012). LEDGF/p75-independent HIV-1 replication demonstrates a role for HRP-2 and remains sensitive to inhibition by LEDGINs. PLoS Pathog.

[37.•] Gijsbers R (2011). Role of the PWWP domain of lens epithelium-derived growth factor (LEDGF)/p75 cofactor in lentiviral integration targeting. J Biol Chem.

[38] Mahmoudi T (2012). The BAF complex and HIV latency. Transcription.

[39] Rafati H (2011). Repressive LTR nucleosome positioning by the BAF complex is required for HIV latency. PLoS Biol.

[40] Arimondo PB (2000). The chromosomal protein HMG-D binds to the TAR and RBE RNA of HIV-1. FEBS Lett.

[41] Hindmarsh P (1999). HMG protein family members stimulate human immunodeficiency virus type 1 and avian sarcoma virus concerted DNA integration in vitro. J Virol.

[42] Lewinski MK (2006). Retroviral DNA integration: viral and cellular determinants of target-site selection. PLoS Pathog.

[43] Christ F (2012). Small-molecule inhibitors of the LEDGF/p75 binding site of integrase block HIV replication and modulate integrase multimerization. Antimicrob Agents Chemother.

[44] Dahabieh MS (2014). Direct non-productive HIV-1 infection in a T-cell line is driven by cellular activation state and NFkappaB. Retrovirology.

[45] Dieudonne M (2009). Transcriptional competence of the integrated HIV-1 provirus at the nuclear periphery. Embo j.

[46] Carteau S, Hoffmann C, Bushman F (1998). Chromosome structure and human immunodeficiency virus type 1 cDNA integration: centromeric alphoid repeats are a disfavored target. J Virol.

[47] Han Y (2008). Orientation-dependent regulation of integrated HIV-1 expression by host gene transcriptional readthrough. Cell Host Microbe.

[48] Duverger A (2009). Determinants of the establishment of human immunodeficiency virus type 1 latency. J Virol.

[49] Lenasi T, Contreras X, Peterlin BM (2008). Transcriptional interference antagonizes proviral gene expression to promote HIV latency. Cell Host Microbe.

[50] Shearwin KE, Callen BP, Egan JB (2005). Transcriptional interference—a crash course. Trends Genet.

[51] Cullen BR, Lomedico PT, Ju G (1984). Transcriptional interference in avian retroviruses—implications for the promoter insertion model of leukaemogenesis. Nature.

[52] Han Y (2004). Resting CD4+ T cells from human immunodeficiency virus type 1 (HIV-1)-infected individuals carry integrated HIV-1 genomes within actively transcribed host genes. J Virol.

[53] Vatakis DN (2009). Human immunodeficiency virus integration efficiency and site selection in quiescent CD4+ T cells. J Virol.

[54] Barr SD (2006). HIV integration site selection: targeting in macrophages and the effects of different routes of viral entry. Mol Ther.

[55] Pierson T, McArthur J, Siliciano RF (2000). Reservoirs for HIV-1: mechanisms for viral persistence in the presence of antiviral immune responses and antiretroviral therapy. Annu Rev Immunol.

[56] Siliciano RF, Greene WC (2011). HIV latency. Cold Spring Harb Perspect Med.

[57] Pierson TC (2002). Intrinsic stability of episomal circles formed during human immunodeficiency virus type 1 replication. J Virol.

[58] Bukrinsky MI (1991). Quiescent T lymphocytes as an inducible virus reservoir in HIV-1 infection. Science.

[59] Spina CA, Guatelli JC, Richman DD (1995). Establishment of a stable, inducible form of human immunodeficiency virus type 1 DNA in quiescent CD4 lymphocytes in vitro. J Virol.

[60] Saleh S (2007). CCR7 ligands CCL19 and CCL21 increase permissiveness of resting memory CD4+ T cells to HIV-1 infection: a novel model of HIV-1 latency. Blood.

[61] Cameron PU (2010). Establishment of HIV-1 latency in resting CD4+ T cells depends on chemokine-induced changes in the actin cytoskeleton. Proc Natl Acad Sci U S A.

[62] Evans VA (2013). Myeloid dendritic cells induce HIV-1 latency in non-proliferating CD4+ T cells. PLoS Pathog.

[63] Shen A (2013). Endothelial cell stimulation overcomes restriction and promotes productive and latent HIV-1 infection of resting CD4+ T cells. J Virol.

[64.•] Pace MJ (2012). Directly infected resting CD4+ T cells can produce HIV Gag without spreading infection in a model of HIV latency. PLoS Pathog.

[65] Brady T (2009). HIV integration site distributions in resting and activated CD4+ T cells infected in culture. AIDS.

[66.••] Sherrill-Mix S (2013). HIV latency and integration site placement in five cell-based models. Retrovirology.

[67.••] Ho YC (2013). Replication-competent noninduced proviruses in the latent reservoir increase barrier to HIV-1 cure. Cell.

[68.••] Maldarelli F (2014). Specific HIV integration sites are linked to clonal expansion and persistence of infected cells. Science.

[69.••] Wagner TA (2014). Proliferation of cells with HIV integrated into cancer genes contributes to persistent infection. Science.

[70] Wei X (1995). Viral dynamics in human immunodeficiency virus type 1 infection. Nature.

[71.•] Imamichi H (2014). Lifespan of effector memory CD4+ T cells determined by replication-incompetent integrated HIV-1 provirus. Aids.

[72] Ikeda T (2007). Recurrent HIV-1 integration at the BACH2 locus in resting CD4+ T cell populations during effective highly active antiretroviral therapy. J Infect Dis.

[73] Gattinoni L (2011). A human memory T cell subset with stem cell-like properties. Nat Med.

[74] Macallan DC (2004). Rapid turnover of effector—memory CD4+ T cells in healthy humans. The Journal of Experimental Medicine.

[75] Lanzavecchia A, Sallusto F (2000). Dynamics of T lymphocyte responses: intermediates, effectors, and memory cells. Science.

[76] Lewinski MK (2005). Genome-wide analysis of chromosomal features repressing human immunodeficiency virus transcription. J Virol.

[77] De Palma M (2005). Promoter trapping reveals significant differences in integration site selection between MLV and HIV vectors in primary hematopoietic cells. Blood.

[78] Liu H (2006). Integration of human immunodeficiency virus type 1 in untreated infection occurs preferentially within genes. J Virol.

